# Clinical application of polar body-based preimplantation genetic testing for maternal mutations in women with a limited number of oocytes

**DOI:** 10.1186/s13023-025-03659-7

**Published:** 2025-04-01

**Authors:** Jia Chen, Xingwu Wu, Qiang Xu, Tao Ding, Ge Chen, Houyang Chen, Yongyi Zou, Jialyu Huang, Ziyu Zhang, Lifeng Tian, Yan Zhao, Ranhui Duan, Zengming Li, Qiongfang Wu, Yanqiu Liu

**Affiliations:** 1 Reproductive Medicine Center, Jiangxi Maternal and Child Health Hospital, 508 West Station Street, Nanchang, Jiangxi 330006 China; 2 Jiangxi Key Laboratory of Reproductive Health, Jiangxi Maternal and Child Health Hospital, Nanchang, Jiangxi 330006 China; 3 Central Laboratory, Jiangxi Maternal and Child Health Hospital, Nanchang, Jiangxi 330006 China; 4 Medical Genetics Center, Jiangxi Maternal and Child Health Hospital, 508 West Station Street, Nanchang, Jiangxi 330006 China; 5 Jiangxi Provincial Clinical Medical Research Center for Obstetrics and Gynecology, Jiangxi Maternal and Child Health Hospital, Nanchang, Jiangxi 330006 China; 6 Center for Medical Genetics, School of Life Sciences, Central South University, Changsha, Hunan 410028 China

**Keywords:** Preimplantation genetic testing, PGT, Monogenic disease, Polar body, A limited number of oocytes, Single nucleotide polymorphism, Linkage analysis

## Abstract

**Background:**

Trophectoderm (TE) cell biopsy at the blastocyst stage is currently the most common method used in preimplantation genetic testing for monogenic disorders (PGT-M). However, this approach may result in the wasting of some genetically unaffected embryos because only a proportion of zygotes develop to the blastocyst stage. Unaffected embryos, which degenerated during blastomere-blastocyst transformation, may give birth if transferred before the blastocyst stage and may be of great value to women with a low oocyte count. This study sought to investigate the potential application of polar-body (PB) biopsy in saving more genetically unaffected embryos for women with disease-causing mutations and a limited number of oocytes during PGT-M.

**Methods:**

Three couples with female partners who had autosomal dominant or X-linked mutations in *IRF6*, *FMR1*, and *EDA* were recruited. The number of retrieved oocytes was limited to six per cycle. The first and second PBs (PB1 and PB2) of each oocyte were biopsied separately and subjected to multiple displacement amplification (MDA). The genotype of each embryo was determined by analyzing the MDA products of the corresponding PB1 and PB2 using a novel approach that combined direct mutation testing and single nucleotide polymorphism linkage analysis. Mutation-free embryos cryopreserved before the blastocyst stage were chosen for transfer.

**Results:**

In total, four cycles were performed, resulting in the retrieval of 15 oocytes for three couples. The genotype of each embryo was successfully determined. Seven mutation-free embryos were discovered. Three of them were transferred, resulting in two clinical pregnancies, and the birth of two healthy infants. The accuracy of the embryo genotypes was validated by genetic testing of fetuses in the second trimester or at birth.

**Conclusions:**

The PB-based strategy is feasible and effective for determining the mutation-carrier statuses of embryos in PGT-M for maternal mutations. Compared to blastocyst stage detection, this method may save a greater number of genetically unaffected embryos for patients. Further clinical trials are needed to determine whether PB biopsy is more beneficial than TE cell biopsy for women with disease-causing mutations and a limited number of oocytes in PGT-M.

## Introduction

Preimplantation genetic testing for monogenic disorders (PGT-M) is an assisted reproductive technology that helps high-risk parents to reduce the chance of having affected babies with genetic disorders by detecting inherited pathogenic mutations in embryos before transfer [1]. PGT-M begins with a biopsy, which can be performed at various stages of embryo development. Polar-body (PB) biopsy is performed at the earliest stage. The first and second PBs (PB1 and PB2) are typically biopsied following oocyte retrieval and fertilization via intracytoplasmic sperm injection (ICSI), respectively. Laser energy is commonly used for zona pellucida opening, while aspiration is used to remove a single PB. PB biopsy can be used to evaluate maternal genetic variants and is currently applied in only a small number of centers [2, 3]. Cleavage-stage embryo biopsy is typically performed on day 3 of preimplantation development. During a blastocyst or trophectoderm (TE) biopsy, the zona pellucida is typically breached on days 3/4 or 5. TE cells are extracted using a combination of aspiration and laser excision from protruding blastocysts or through aspiration and mechanical dissection from blastocysts on day 5/6.

Presently, TE biopsy is the most common technique used in PGT procedures [3]. In comparison to cleavage-stage embryo biopsy, TE biopsy provides two significant advantages. First, cleavage-stage biopsy significantly reduces the possibility of human embryonic implantation, whereas TE biopsy does not. The sustained implantation rate of biopsied blastomeres on day 3 was found to be approximately 39% lower than in unbiopsied controls. In contrast, similar sustained implantation rates were observed in biopsied and unbiopsied blastocysts [4]. The decreased susceptibility of blastocysts to potential embryo damage may be caused by the preservation of the inner cell mass from which the fetus develops. Additionally, more cells (ideally five to eight cells) can be biopsied at the blastocyst stage, whereas only one cell should be removed at the cleavage stage. A two-cell removal at the cleavage stage was found to be more harmful to embryonic development and implantation potential than a single-cell removal [5]. The damage caused by TE biopsy was largely determined by the TE quality and experience of the various embryologists. When less than 41 cells were biopsied, increasing the number of biopsied TE cells had no significant effect on the survival and implantation rates of blastocysts with a grade A TE score. However, having more biopsied cells increases the accuracy of genetic analysis [6]. These benefits contribute to the widespread use of TE biopsy as the preferred method for collecting samples from preimplantation embryos in PGT.

A primary concern for patients undergoing TE biopsy in PGT is the low rate of blastocyst formation. Several factors influenced blastocyst development, including female age, body mass index (BMI), number of retrieved oocytes, fertilization method, embryo culture conditions, blastomere count, and embryo quality. Reported blastocyst formation rates typically range from 40 to 60% [7–9]. TE biopsy may result in the loss of some embryos, because not all embryos progress to the blastocyst stage. The genetically unaffected embryos that degenerate during blastomere-blastocyst transformation have the potential to give birth if transferred. These discarded embryos may be extremely valuable for women with disease-causing mutations and a limited number of oocytes in PGT-M cycles.

PB biopsy is a safe and effective method for detecting maternal genetic variants in PGT [10]. PBs are by-products of oocyte meiosis and are not required for successful fertilization or normal embryonic development. PB1 has a subset of bivalent chromosomes after homologous chromosome separation in the first meiosis, whereas PB2 has a haploid set of chromatids after the separation of sister chromosome monomers in the second meiosis. Detecting PB1 and PB2 could help determine whether the embryo carries the maternal mutation [11]. PB biopsy may be more beneficial for women with a low number of oocytes in PGT-M. In comparison to TE biopsy, PB biopsy has the potential to save a greater number of genetically unaffected embryos and improve oocyte utilization by determining mutation-carrier status before blastocyst formation. Additionally, PB biopsy is less harmful to embryos than blastomere biopsy. These advantages make PB biopsy a more promising option.

**Fig. 1:**
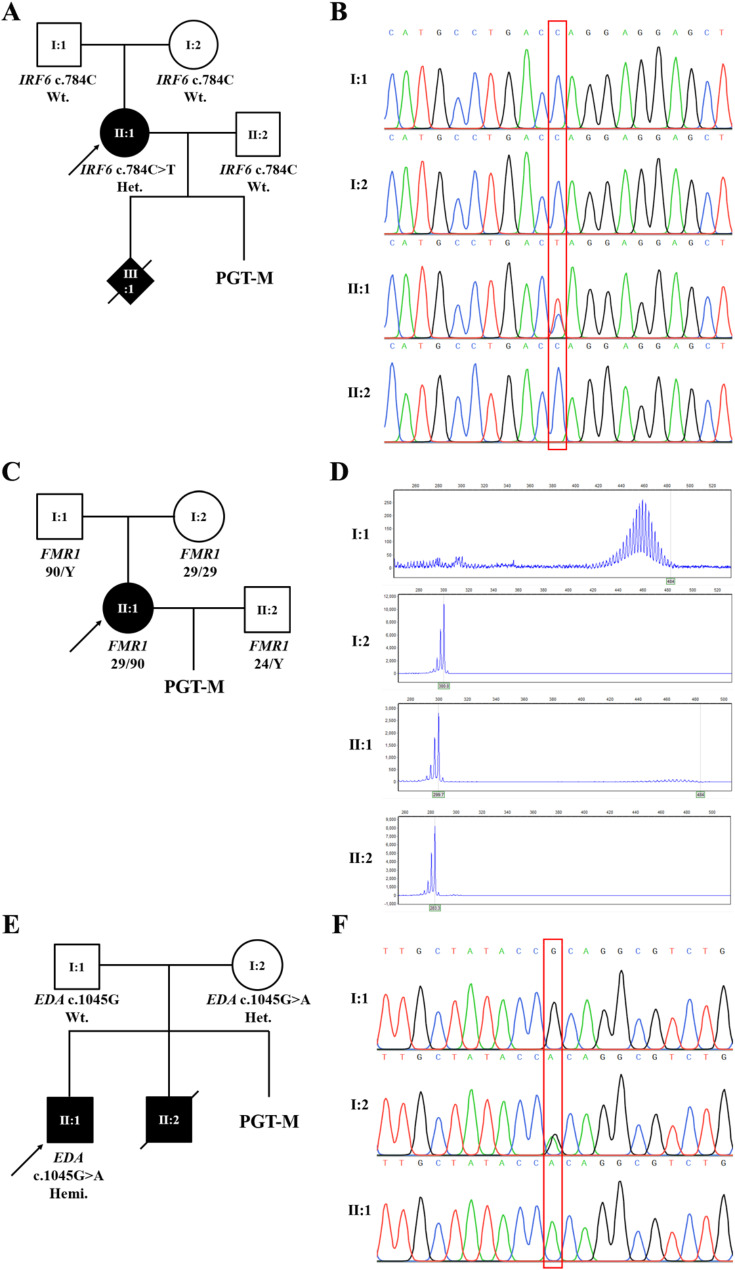
Workflow of analysis. Women carrying with autosomal dominant or X-linked mutations and the number of expected retrieved oocytes ≤ 6 were recruited in the study. The mutations were detected in the family members. For female partners with inherited mutations, genotyped family members were selected to linkage analysis to get the haplotypes by targeted sequencing SNPs flanking the mutations. Then controlled ovarian hyperstimulation was conducted in the women. PB1 and PB2 of each oocyte were biopsied respectively following oocyte retrieval and ICSI. The zygotes were cultured in vitro and the embryos were vitrified before blastocyst stage. The mutation-carrier status of each embryo was deduced by direct mutation detection and haplotype analysis in the corresponding PB1 and PB2. The mutation-free embryos were selected to FET. For female partners with *de novo* mutations, the only difference was that the haplotypes were obtained by linkage analysis of the women and PBs after genotyping by direct mutation detection

In this study, a novel PB biopsy-based strategy (Fig. 1) was used to perform PGT-M on three couples with women carrying disease-causing mutations in autosomal dominant (*IRF6*) or X-linked monogenic genes (*FMR1* and *EDA*). Each woman had ≤ 6 retrieved oocytes per cycle. Using this strategy, two of the three families delivered healthy babies. This study demonstrated the efficacy of using PB1 and PB2 to detect maternal mutations in PGT-M, indicating potential benefits for women with a limited number of oocytes.

## Materials and methods

### Patients

Three couples, with the female partners carrying autosomal dominant or X-linked monogenic disease-causing mutations, were recruited from the Reproductive Medicine Center of Jiangxi Maternal and Child Health Hospital. The study ran from August 2020 to July 2023.

**Fig. 2:**
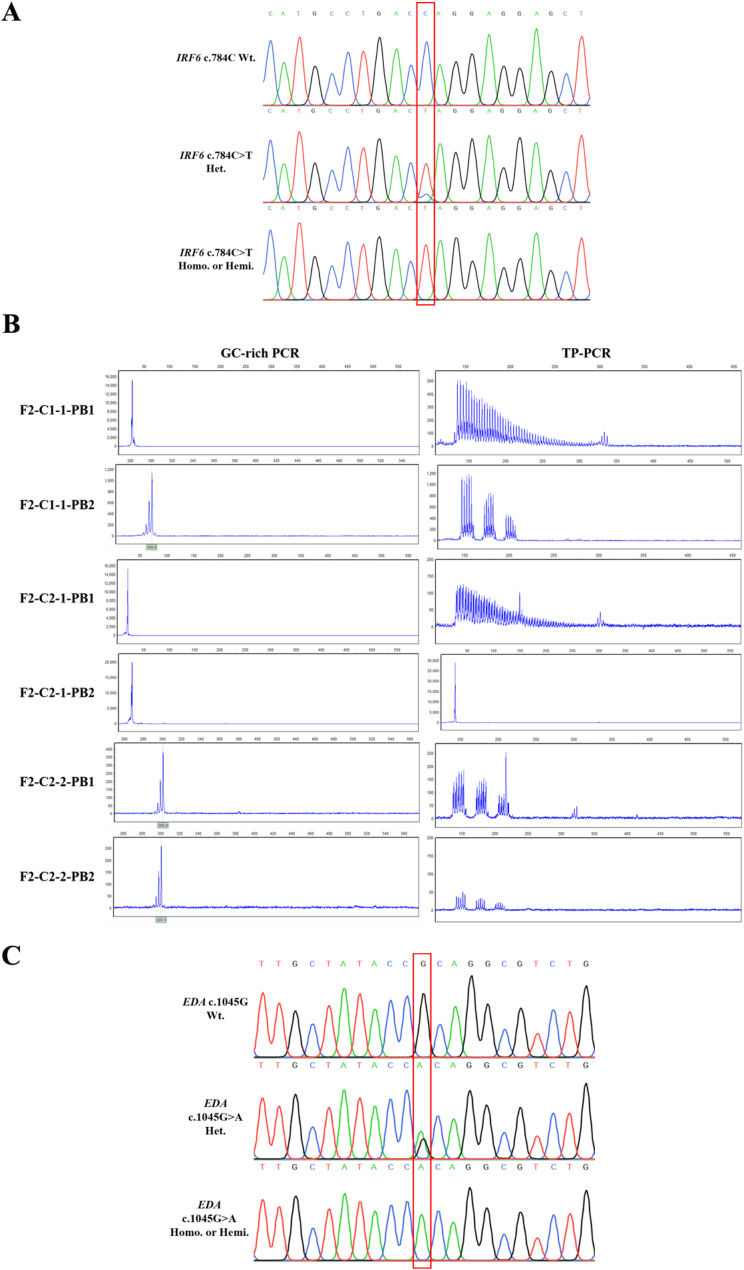
Pedigrees and mutation detection results in family members. **A**: The pedigree and genotypes of family members in case (1) **B**: Sanger sequence chromatograms of the family members in case 1 reveals the proband carrying a *de novo* mutation of *IRF6* c.784 C > T. **C**: The pedigree and the number of *FMR1* CGG repeats of family members in case (2) **D**: Fragment length analysis of GC-rich PCR products of indicating the number of *FMR1* CGG repeats of family members in case 2. **E**: The pedigree and genotypes of family members in case (3) **F**: Sanger sequence chromatograms of the family members in case 3 reveals the proband carrying an inherited mutation of *EDA* c.1045G > A. Open symbols, unaffected; filled symbols, affected; squares, male; circles, female; rhombuses, unkown gender; arrow, the proband; symbols with diagonals, death

In Case 1, the patient (II:1, Fig. 2A) was a 33-year-old woman with congenital orofacial clefts. No discernible phenotypic abnormalities were found in her parents or spouse. Her husband denied a family history of genetic disorders. After conceiving a fetus with cleft lip and palate, the couple decided to end the pregnancy. Trio whole exome sequencing (WES) of the proband and her parents indicated a *de novo* variant c.784 C > T (p.Gln262*) in *IRF6* (NM_006147.3). The proband’s husband’s WES data contained no suspected variants associated with orofacial cleft. Sanger sequencing of the family members confirmed the *de novo* variant (Fig. 2B). The *IRF6* gene, which encodes interferon regulatory transcription factor 6, is linked to Van der Woude Syndrome (VWS), an autosomal dominantly inherited developmental disorder characterized by cleft lip and/or palate. The *IRF6* c.784 C > T variant was found in a patient with VWS [12]. According to the American College of Medical Genetics and Genomics (ACMG) guidelines’ recommendation on sequence variants interpretation [13], the *IRF6* c.784 C > T variant would be classified as “pathogenic,” meeting the criteria PVS1 + PS2_Supporting + PM2_Supporting, and was identified as the proband’s disease-causing factor. The PVS1 was used at very strong level because the variant is nonsense and predicted to undergo nonsense-mediated decay, loss of function is a known mechanism of the disease, and the involved exon is present in biologically-relevant transcript. The PS2 was used at supporting level considering the variant was *de novo* in the proband with no family history. The PM2 was used at supporting level because the variant was absent in the gnomAD population databases (http://gnomad-sg.org/).

In Case 2, the patient (II:1, Fig. 2C) was a 32-year-old woman suffering from premature ovarian failure (POF). Her follicle-stimulating hormone level was 59.32 mIU/mL, while anti-mullerian hormone was 0.052ng/ml. The fragile X mental retardation 1 gene (*FMR1*), the most common disease-causing gene for POF, was tested. She was identified as a premutation carrier of *FMR1* with CGG repeats of 29 and 90. It could be concluded that she had a high risk of having a child with fragile X syndrome. She and her husband chose PGT-M to avoid having an affected baby. *FMR1* CGG repeats for the participant’s father, mother, and husband were 90/Y, 29/29, and 24/Y, respectively (Fig. 2C, D).

In Case 3, the female participant (I:2, Fig. 2E) was 31 years of age. She and her husband were unaffected, but they had two boys with hypohidrotic ectodermal dysplasia (HED). The little son had died. A hemizygous missense variant c.1045G > A (p.Ala349Thr) of the *EDA* gene (NM_001399.5) was discovered in the proband (II:1, Fig. 2E) by Trio-WES. Defects in the *EDA* gene result in X-linked HED, which is characterized by hypotrichosis, hypohidrosis, and hypodontia. The *EDA* c.1045G > A (p.Ala349Thr) variant was recorded as “pathogenic” in the ClinVar (https://www.ncbi.nlm.nih.gov/clinvar/, Variation ID: 11040) and was also classified as pathogenic based on the ACMG guidelines by meeting the criteria PM6_Strong (3 HED patients were reported carrying the variant in *de novo* inheritance [14–16]), PS4_Moderate (several HED patients were reported carrying the variant [17–19]), PP3_Moderate (REVEL score 0.887), PM2_Supporting (absent in the gnomAD databases), and PP1 (the variant was co-segregated with the disease). Sanger sequencing of family members revealed that the proband’s mother had the heterozygous variant of *EDA* c.1045G > A (Fig. 2E, F).

Poor ovarian reserve was a common symptom among all three female participants. A total of ≤ 6 antral follicles in bilateral ovaries detected by transvaginal ultrasonography on the second day of menstruation indicated poor ovarian reserve in our study. Following genetic counseling, all three couples refused to transfer embryos with disease-causing mutations and agreed to participate in our PGT-M study via PB biopsy. Cases 1 and 3were treated with PGT-M once, while Case 2 was treated twice. All couples provided written informed consent during their initial consultation. The study was authorized by the Ethics Committee of Jiangxi Maternal and Child Health Hospital.

### Genetic variation detection

Genomic DNA was extracted from peripheral blood samples with the QIAamp DNA Blood Mini kit (QIAGEN, Germany). Trio-based and proband-only WES approaches were used to determine genetic mutations in cases 1 and 3, respectively. The sequencing and analysis protocols were carried out according to established methods [20]. Candidate mutations were identified in the probands and confirmed in other family members using Sanger sequencing. The pathogenic criteria for the detected mutations were interpreted using the ACMG guidelines [13] and ClinVar (https://www.ncbi.nlm.nih.gov/clinvar/). In case 2, the *FMR1* CGG repeats were quantified using GC-rich PCR and Triplet-primed (TP) PCR, as described by Gao et al. 2020 [21]. The GC-rich PCR was used to directly determine CGG repeat counts based on allele size, whereas the TP-PCR allowed for qualitative detection of the expanded allele [22].

### Workflow of analysis

The workflow for the strategy is illustrated in Fig. 1. Initially, disease-causing mutations were found in family members. Subsequently, for female partners with inherited mutations, single nucleotide polymorphisms (SNPs) flanking the mutation were evaluated in the genomic DNA of selected family members. Linkage analysis was used to determine the haplotype of the chromosome segment carrying the mutation. For women with *de novo* mutations, each oocyte’s PB1 and PB2 were biopsied. Individual PBs underwent whole genome amplification (WGA) using multiple displacement amplification (MDA) methodology. The mutation was detected directly in each PB’s MDA product. The SNPs surrounding the mutation were examined in the genotyped proband and PBs to determine the haplotype. After oocyte fertilization and PB biopsy, the embryos were cryopreserved using vitrification on Day 3. The mutation-carrier status of the corresponding embryo was determined through direct mutation detection and haplotyping analysis of the PB1 and PB2. Embryos without the mutation were selected for frozen-thawed embryo transfer (FET).

### Controlled ovarian hyperstimulation and oocyte retrieval

Controlled ovarian hyperstimulation was performed using flexible gonadotropin-releasing hormone antagonist regimen. Briefly, patients were injected daily with 150–300 IU recombinant follicle-stimulating hormone (FSH; Gonal-F, Merck Serono, Switzerland) intramuscularly from menstrual cycle day 2 onward. The starting FSH dose was determined by the patient’s age, BMI and ovarian reserve, with subsequent adjustment according to follicular monitoring by transvaginal ultrasound and serum hormone measurement. When the leading follicle size reached 14 mm, cetrorelix acetate (Cetrotide, Merck Serono, Switzerland) was added at a dose of 0.25 mg per day. Final oocyte maturation was co-triggered with 250 µg recombinant human chorionic gonadotropin (Ovidrel, Merck Serono, Switzerland) and 0.1 mg triptorelin (Decapeptyl, Ferring Pharmaceuticals, Switzerland), as soon as the follicles were ≥ 18 mm. Transvaginal ultrasound-guided oocyte retrieval was scheduled 36 to 38 h later.

### PB biopsy

In vitro fertilization via ICSI was utilized to fertilize the oocytes. Biopsies of PB1 were performed 2–3 h after oocyte retrieval with laser assistance, and biopsies of PB2 were performed 5–6 h after ICSI.

### Single PB WGA and mutation detection

The single PB was treated with MDA (Qiagen, REPLI-g Single Cell Kit) for WGA. The resulting MDA DNA was diluted fivefold with nuclease-free ultra-pure water to detect mutations. The methods used for detecting mutations in blood-derived genomic DNA were also applied to MDA DNA obtained from PB samples.

### Haplotype analysis

SNPs with minor allele frequencies 0.3–0.5 were chosen within 2 Mb upstream and downstream of the *IRF6*, *FMR1*, and *EDA* gene mutations, respectively. The primers for the SNPs were designed and synthesized (Twist Bioscience, USA). DNA samples from family members and MDA products of PBs were subjected to multiplex PCR with the targeted primers. The amplified DNA was purified using XP beads (Thermo Fisher Scientific, USA) and the concentrations of each DNA sample were determined. The library was prepared with 200ng purified DNA using the AmpSeq Library Prep Kit V2 (Vazyme Biotech, Nanjing, China). The library was then sequenced using the Miseq Dx sequencer (Illumina, USA). In case 1, the genomic DNA of the proband and the MDA products of the selected PBs were subjected to targeted SNP amplification following successful genotyping via Sanger sequencing. The products of SNP amplification were then sequenced to obtain information about the SNPs. The heterozygous SNPs discovered in the proband were considered effective SNPs and used for haplotype analysis. Linkage analysis was used to determine the haplotype associated with the *IRF6* mutation. In cases 2 and 3, genomic DNA from selected genotyped family members was used to identify haplotypes via targeted SNP sequencing. Specific PBs were chosen for haplotyping. The embryos’ mutation-carrier statuses were inferred and validated using the haplotypes of PB1 and PB2 derived from each oocyte.

### FET and validation of the results

Mutation-free embryos were chosen for transfer in FET cycles. The accuracy of PGT-M was confirmed through a genetic test of amniotic fluid at 18 gestational weeks or umbilical cord blood at birth.

## Results

### Direct mutation detection in PBs

**Fig. 3:**
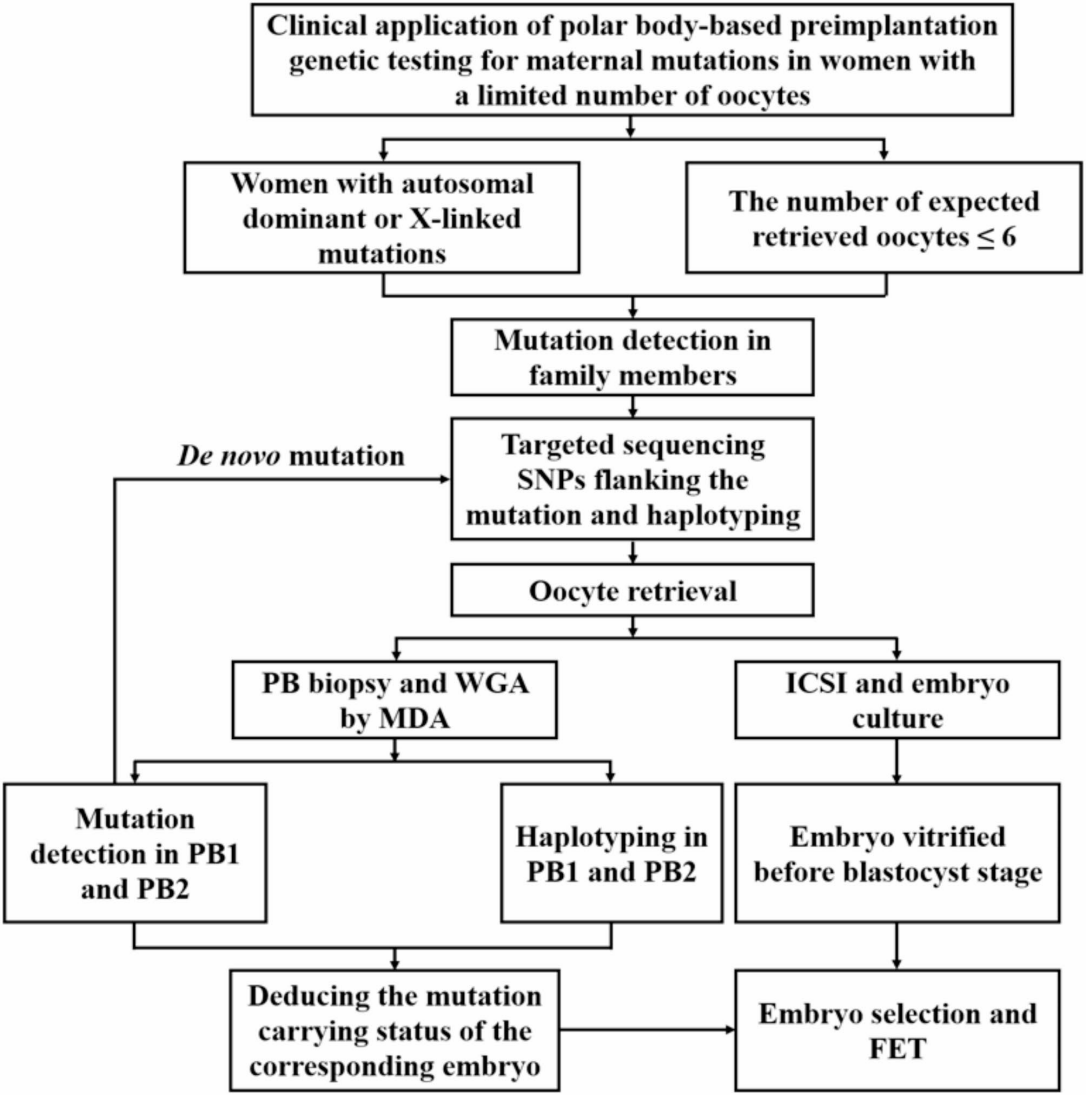
Direct mutation detection results of PBs. **A**: Sanger sequence chromatograms of *IRF6* c.784 of PBs in case (1) **B**: GC-rich and TP-rich PCR results of *FMR1* CGG mutation of PBs in case (2) **C**: Sanger sequence chromatograms of *EDA* c.1045 of PBs in case 3

In total, 15 PB1s/PB2s from 4 PGT-M cycles were biopsied in three couples. The mutation-carrier statuses of the corresponding embryos were deduced from the test results of PB1s and PB2s. The detection chromatograms were shown in Fig. 3 and the results were presented in Table 1.

**Table 1:** Direct detection results of the disease-causing mutations in Polar bodies

Case No.	Maternal mutations	Oocyte retrieval cycle No.	Oocyte No.	Cell type (Sample NO.)	Direct detection results of the variants in polar bodies	Deduced risk of embryos carrying the mutations	The number of low-risk embryos
1	*IRF6* c.784 C > T Het.	1	F1-1	PB1 (F1-1-PB1)	Wt. (Mutant allele drop-out)	High	2
				PB2 (F1-1-PB2)	Wt.		
			F1-2	PB1 (F1-2-PB1)	*IRF6* c.784 C > T Homo.	Uncertain (Probably Low)	
				PB2 (F1-2-PB2)	Wt.		
			F1-3	PB1 (F1-3-PB1)	*IRF6* c.784 C > T Het.	High	
				PB2 (F1-3-PB2)	Wt.		
			F1-4	PB1 (F1-4-PB1)	Wt.	Uncertain (Probably High)	
				PB2 (F1-4-PB2)	*IRF6* c.784 C > T Hemi.		
			F1-5	PB1 (F1-5-PB1)	*IRF6* c.784 C > T Het.	High	
				PB2 (F1-5-PB2)	Wt.		
			F1-6	PB1 (F1-6-PB1)	*IRF6* c.784 C > T Homo.	Uncertain (Probably Low)	
				PB2 (F1-6-PB2)	Wt.		
2	*FMR1* CGG repeat number 29/90	1	F2-C1-1	PB1 (F2-C1-1-PB1)	Mutant allele Homo.	Low	1
				PB2 (F2-C1-1-PB2)	Normal allele Hemi.		
		2	F2-C2-1	PB1 (F2-C2-1-PB1)	Mutant allele Homo.	Low	1
				PB2 (F2-C2-1-PB2)	Detection failure		
			F2-C2-2	PB1 (F2-C2-2-PB1)	Normal allele Homo. (Mutant allele drop-out)	High	
				PB2 (F2-C2-2-PB2)	Normal allele Hemi.		
3	*EDA* c.784 C > T Het.	1	F3-1	PB1 (F3-1-PB1)	*EDA* c.1045G > A Homo.	Uncertain (Probably Low)	3
				PB2 (F3-1-PB2)	Detection failure		
			F3-2	PB1 (F3-2-PB1)	Wt. (Mutant allele drop-out)	High	
				PB2 (F3-2-PB2)	Wt.		
			F3-3	PB1 (F3-3-PB1)	Wt.	Uncertain (Probably High)	
				PB2 (F3-3-PB2)	*EDA* c.1045G > A Hemi.		
			F3-4	PB1 (F3-4-PB1)	*EDA* c.1045G > A Homo.	Uncertain (Probably Low)	
				PB2 (F3-4-PB2)	Wt.		
			F3-5	PB1 (F3-5-PB1)	*EDA* c.1045G > A Het.	High	
				PB2 (F3-5-PB2)	Wt.		
			F3-6	PB1 (F3-6-PB1)	*EDA* c.1045G > A Homo.	Uncertain (Probably Low)	
				PB2 (F3-6-PB2)	Wt.		

In Case 1, 6 oocytes were retrieved in single cycle. PB1 and PB2 of each oocyte were biopsied and Sanger sequenced (Fig. 3A; Table 1). In oocyte 1, only the wildtype genotype was found in PB1 (F1-1-PB1) and PB2 (F1-1-PB2), indicating that the corresponding embryo carried the mutant allele. The F1-1-PB1 should have one wildtype allele and one mutant allele. The absence of the mutant allele in F1-1-PB1 indicates that the mutant allele was dropped out during detection. PBs from oocytes 3 and 5 performed similarly to PBs from oocyte 1, with the exception of the absence of allele drop-out (ADO) in the detection of PB1s (F1-3-PB1 and F1-5-PB1). In oocytes 2 and 6, the homozygous mutant genotype was detected in the PB1s (F1-2-PB1 and F1-6-PB1), while the wildtype was detected in the PB2s (F1-2-PB2 and F1-6-PB2). The mutation-carrier statuses of these embryos were ambiguous due to the possibility of ADO during the detection of the PB1s. The ADO rate for detecting genetic markers in PB1 was reported to be between 5.9% and 9.6% [23]. Given the low ADO rate, the corresponding embryos from oocytes 2 and 6 most likely inherited the wildtype allele. A similar approach was used to determine the mutation-carrier status of the embryo from oocyte 4, which indicated that it most likely carried the mutant allele.

In Case 2, three oocytes were collected from two cycles. The CGG mutation of *FMR1* was detected in PBs using GC-rich PCR and TP-PCR (Fig. 3B; Table 1). In the first cycle, only one oocyte was retrieved. Only the expanded CGG allele was detected in the PB1 (F2-C1-1-PB1) by TP-PCR, indicating that it carried the homozygous *FMR1* CGG mutant allele, and no ADO occurred during the detection. Because it was unlikely that the expanded allele would be retained while the unexpanded allele was dropped out. The failure of GC-rich PCR to directly detect the CGG repeat in PB1 (F2-C1-1-PB1) was primarily due to the large size of the expanded allele, which could not be amplified by the MDA technique. The unexpanded CGG allele was found in the PB2 (F2-C1-1-PB2) by both GC-rich PCR and TP-PCR. These results indicated that the corresponding embryo inherited the normal *FMR1* allele. In the second cycle, two oocytes were retrieved. Analysis of the detection results of PB1s and PB2s (Fig. 3B; Table 1) revealed that the embryo from oocyte 1 (F2-C2-1) had a normal allele, whereas the embryo from oocyte 2 (F2-C2-2) had an expanded allele. The failure to detect PB2 in oocyte 1 could have been due to DNA degradation during the biopsy or an amplification failure during the MDA procedure.

In Case 3, six oocytes were retrieved in a single cycle. Similar detection and analysis methods were used (Fig. 3C; Table 1). The embryos from oocytes 2 and 5 were found to carry the mutant allele, while the mutation-carrier statuses of the remaining embryos were unknown. Specifically, embryos from oocytes 1, 4, and 6 were likely to inherit the normal allele, whereas the embryo from oocyte 3 was likely to carry the mutant allele, assuming the absence of ADO.

### Haplotype analysis of family members and PBs

The PBs of which the corresponding embryos’ mutation-carrier statuses were uncertain in Table 1 and specific family members were chosen to conduct linkage analysis to assess whether ADO had affected the PBs’ direct mutation detection in Cases 1 and 3. Table 2 showed the SNP haplotypes of the proband and PBs from oocytes F1-2 and F1-4 in Case 1. The haplotypes of the proband, the proband’s mother, and PBs from oocytes F3-1, F3-3, and F3-4 in Case 3 were presented in Table 4. The PB1s and PB2s from oocytes F1-6 and F3-6 were discovered to share the same haplotypes with the corresponding PBs from oocyte F1-2 and F3-4. Their haplotypes were not shown for avoiding redundancy. The haplotypes of detected PBs were found to be consistent with the results obtained through direct mutation detection. These findings indicated that no ADO affected the direct mutation detection results, the embryos from oocytes F1-2, F1-6, F3-1, F3-4, and F3-6 inherited the wildtype alleles, whereas the embryo from oocytes F1-4 and F3-3 carried the mutant alleles. In Case 2, haplotyping was performed on the proband, the proband’s father, and the corresponding PBs of low-risk embryos (PBs from oocytes F2-C1-1 and F2-C2-1) to validate the mutation detection findings in the PBs (Tables 1 and 3). The haplotypes and direct mutation detection results of PBs were consistent, confirming the low pathogenicity of the embryos from oocytes F2-C1-1 and F2-C2-1.

**Table 2:** The SNP haplotypes flanking the *IRF6* gene (chr1:209,958,968 − 209,979,520) in case 1

Chromosome	SNP position (GRCH37/hg19)	Proband (II:1)		Case 1			Oocyte F1-4		
				Oocyte F1-2					
				F1-2-PB1		F1-2-PB2	F1-4-PB1		F1-4-PB2
Chr1	209,656,197	**A**	**G**	**A**	**A**	**G**	**G**	**G**	**A**
Chr1	209,663,226	**C**	**G**	**C**	**C**	**G**	**G**	**G**	**C**
Chr1	209,700,808	**T**	**A**	**T**	**T**	**A**	**A**	**A**	**T**
Chr1	209,712,889	**T**	**C**	**T**	**T**	**?**	**C**	**C**	**T**
Chr1	209,887,369	**C**	**G**	**C**	**C**	**G**	**G**	**G**	**C**
Chr1	209,965,556	**A**	**T**	**A**	**A**	**T**	**T**	**T**	**A**
Chr1	210,000,282	**C**	**T**	**C**	**C**	**T**	**T**	**T**	**C**
Chr1	210,005,713	**T**	**C**	**T**	**T**	**C**	**C**	**C**	**T**
Chr1	210,009,372	**A**	**G**	**A**	**A**	**G**	**G**	**G**	**A**
Chr1	210,020,971	**T**	**C**	**T**	**T**	**C**	**C**	**C**	**T**
Chr1	210,044,393	**C**	**G**	**C**	**C**	**G**	**G**	**G**	**C**
Chr1	210,104,598	**A**	**G**	**A**	**A**	**?**	**G**	**G**	**A**
Chr1	210,134,382	**A**	**G**	**A**	**A**	**G**	**?**	**?**	**A**
Chr1	210,176,904	**A**	**G**	**A**	**A**	**G**	**G**	**G**	**A**
Chr1	210,194,685	**A**	**G**	**A**	**A**	**G**	**G**	**G**	**?**

Note: The deep orange marked haplotype indicates the chromosome segment carrying the mutant allele (*IRF6* c.784 C > T) and the light orange marked haplotype indicates the chromosome segment carrying the normal allele. “?” indicates the genotype of the SNP was detected failure

**Table 3:** The SNP haplotypes flanking the *FMR1* gene (chrX:146,993,437 − 147,032,645) in case 2

Case 2										
Chromosome	SNP position (GRCH37/hg19)	Proband (II:1)		The proband’s father (I:1)	Oocyte 1 in cycle 1 (F2-C1-1)			Oocyte 1 in cycle 2 (F2-C2-1)		
					F2-C1-1-PB1		F2-C1-1-PB2	F2-C2-1-PB1		F2-C2-1-PB2
ChrX	146,680,236	**T**	**C**	**T**	**T**	**T**	**?**	**T**	**T**	**?**
ChrX	146,683,786	**C**	**T**	**C**	**C**	**C**	**T**	**C**	**C**	**?**
ChrX	146,684,940	**T**	**C**	**T**	**T**	**T**	**C**	**T**	**T**	**?**
ChrX	146,689,817	**G**	**A**	**G**	**G**	**G**	**A**	**G**	**G**	**?**
ChrX	146,919,520	**T**	**G**	**T**	**T**	**T**	**G**	**T**	**T**	**?**
ChrX	146,958,977	**T**	**C**	**T**	**T**	**T**	**C**	**T**	**T**	**?**
ChrX	147,401,224	**C**	**G**	**C**	**C**	**C**	**G**	**C**	**C**	**?**
ChrX	147,746,704	**G**	**C**	**G**	**G**	**G**	**C**	**G**	**G**	**?**
ChrX	147,945,641	**A**	**G**	**A**	**A**	**A**	**G**	**A**	**A**	**?**
ChrX	148,017,272	**A**	**G**	**A**	**A**	**A**	**G**	**A**	**A**	**?**
ChrX	149,497,073	**A**	**G**	**A**	**A**	**A**	**G**	**A**	**A**	**?**
ChrX	149,497,116	**C**	**T**	**C**	**C**	**C**	**T**	**C**	**C**	**?**

Note: The deep orange marked haplotype indicates the chromosome segment carrying the mutant allele (*FMR1* CGG_90_) and the light orange marked haplotype indicates the chromosome segment carrying the normal allele. “?” indicates the genotype of the SNP was detected failure

**Table 4:** The SNP haplotypes flanking the *EDA* gene (chrX:68,835,957 − 69,259,322) in case 3

Chromosome	SNP position (GRCH37/hg19)	The proband’s mother (I:2)		Proband (II:1)	Case 3			Oocyte F3-3			Oocyte F3-4		
					Oocyte F3-1								
					F3-1-PB1		F3-1-PB2	F3-3-PB1		F3-3-PB2	F3-4-PB1		F3-4-PB2
ChrX	68,513,890	**G**	**A**	**G**	**G**	**G**	**?**	**A**	**A**	**G**	**G**	**G**	**A**
ChrX	68,556,523	**T**	**C**	**T**	**T**	**T**	**?**	**C**	**C**	**?**	**T**	**T**	**C**
ChrX	68,556,528	**A**	**G**	**A**	**A**	**A**	**?**	**G**	**G**	**A**	**A**	**A**	**?**
ChrX	68,770,723	**T**	**C**	**T**	**T**	**T**	**?**	**C**	**C**	**T**	**T**	**T**	**C**
ChrX	68,777,145	**G**	**T**	**G**	**G**	**G**	**?**	**T**	**T**	**G**	**?**	**?**	**T**
ChrX	68,790,706	**G**	**A**	**G**	**G**	**G**	**?**	**A**	**A**	**G**	**G**	**G**	**A**
ChrX	69,266,212	**G**	**A**	**G**	**G**	**G**	**?**	**A**	**A**	**G**	**G**	**G**	**A**
ChrX	69,271,943	**T**	**C**	**T**	**T**	**T**	**?**	**C**	**C**	**T**	**T**	**T**	**C**
ChrX	69,288,187	**A**	**C**	**A**	**A**	**A**	**?**	**C**	**C**	**A**	**A**	**A**	**?**
ChrX	69,291,656	**A**	**G**	**A**	**A**	**A**	**?**	**G**	**G**	**A**	**A**	**A**	**G**
ChrX	69,308,348	**T**	**C**	**T**	**T**	**T**	**?**	**C**	**C**	**T**	**T**	**T**	**C**
ChrX	69,409,848	**T**	**C**	**T**	**T**	**T**	**?**	**C**	**C**	**?**	**T**	**T**	**C**
ChrX	69,423,721	**T**	**C**	**T**	**T**	**T**	**?**	**C**	**C**	**T**	**T**	**T**	**C**
ChrX	69,440,241	**A**	**G**	**A**	**A**	**A**	**?**	**G**	**G**	**A**	**A**	**A**	**G**

Note: The deep orange marked haplotype indicates the chromosome segment carrying the mutant allele (*EDA* c.1045G > A) and the light orange marked haplotype indicates the chromosome segment carrying the normal allele. “?” indicates the genotype of the SNP was detected failure

### Embryo selection and transfer

The mutation-free embryos identified by direct mutation detection and haplotype analysis were chosen for transfer. According to the cleavage-stage embryo morphology assessment criteria published in the Istanbul consensus [24], embryos from oocytes F1-2, F2-C1-1, and F2-C2-1 were scored as Grade B, while those from oocytes F1-6, F3-1, F3-4, and F3-6 were scored as Grade C. Case 1 chose the embryo from oocyte F1-2, Case 2 chose the embryo from oocyte F2-C2-1, and Case 3 chose the embryo from oocyte F3-1 for the first transfer. Cases 1 and 2 involved couples who were clinically pregnant, whereas Case 3 did not.

### Validation of the PGT-M results

In Case 1, ultrasound methods detected no cleft lip or palate in the fetus during the pregnancy. A healthy baby girl was born, and the *IRF6* c.784 C > T mutation was not found in the genomic DNA of the umbilical cord blood at birth (data not shown). In Case 2, the couple underwent an amniocentesis at 18 weeks of gestation. The PGT-M result was reconfirmed by detecting genomic DNA mutations in amnion cells (data not shown). A healthy baby girl was born after a normal gestation period. Case 3 was awaiting another embryo transfer cycle.

## Discussion

When conducting PGT-M for maternal mutations, PB-based procedure is an alternative approach to TE biopsy and may preserve potential benefit from the viewpoint of saving more unaffected transferable embryos. To increase the availability of transferable embryos for women with low oocyte reserves, we used a novel PB-based testing strategy to perform PGT-M on three couples whose female partners carried pathogenic mutations. In Case 1, six oocytes were retrieved from a female proband with autosomal dominant orofacial clefts caused by the *IRF6* c.784 C > T mutation, and two unaffected embryos were found. The female patient in Case 2 was diagnosed with premature ovarian failure due to the *FMR1* premutation and underwent two cycles, resulting in the retrieval of three oocytes and the preservation of two unaffected embryos. In Case 3, three of the six embryos did not carry the *EDA* c.1045G > A mutation in the woman who had previously given birth to two boys with X-linked HED. The PB-based method accurately deduced each oocyte’s mutation-carrier status, allowing for the maximum retention of genetically unaffected embryos. The patients in Cases 1 and 2 became pregnant and gave birth to two healthy baby girls following the transfer of one of the unaffected embryos for each couple. The fact that the patient in Case 3 did not become pregnant could be attributed to the embryo transferred in Case 3 being Grade C, whereas the embryos transferred in Cases 1 and 2 were Grade B.

To ensure the accuracy of the deduction, a methodology that combined mutation detection and haplotype analysis was used to analyze PBs. The use of PB-based testing in PGT-M was firstly documented in 1990 [25]. Initially, only PB1 was directly tested for mutations, allowing the oocyte’s mutation-carrier status to be determined in cases where a homozygous genotype was identified [25]. However, this PB1 approach could not predict the oocyte’s eventual genotype in the case of a heterozygous genotype, and the potential impact of ADO on PB1 detection results remained unknown. The polymorphic markers flanking mutations were then tested in PB1 and PB2 [11, 26]. A large experience of 938 PB-based PGT-M cycles performed by simultaneously detecting the mutation and polymorphic markers for 146 different monogenic diseases revealed an extremely high accuracy rate of more than 99% [11]. In our study, a similar methodology was used with some modifications, such as using SNPs instead of short tandem repeats as polymorphic markers. This change was made in order to potentially improve the efficacy of identifying a sufficient number of informative markers, as targeted next-generation sequencing can detect hundreds of SNPs at once. Furthermore, haplotype analysis was performed only when a homozygous genotype of PB1 was detected, and an opposite genotype was detected in PB2 (or when genotype detection failed in PB2) (Tables 1, 2, 3 and 4). Given that direct mutation testing using Sanger sequencing or GC-PCR/TP-PCR is much less expensive than haplotype analysis by targeted next-generation sequencing, our testing strategy of applying haplotype analysis to the PBs from genotype uncertain embryos screened by direct mutation test would reduce testing expenses for patients when compared to applying haplotype analysis to all PBs. Due to the failure to detect CGG repeat sizes in the PB1s (F2-C1-1-PB1 and F2-C2-1-PB1) (Fig. 3B), haplotype analysis was used in these samples to validate the inference.

PGT-M is difficult to be performed in women with premature ovarian failure caused by *FMR1* premutation. Aside from poor oocyte reservation, the other challenge is determining the CGG expansion status of *FMR1* alleles in preimplantation samples. Linkage analysis by polymorphic markers was commonly used to trace the risk allele’s transmission [27]. However, the efficacy of this approach is dependent on the availability of informative markers and their proximity to the *FMR1* CGG repeat. Reliability is called into question when there are few informative markers in the designated region. This deficiency could be addressed by directly detecting the *FMR1* CGG repeat. In our study, we used GC-rich PCR and TP-PCR to directly test the *FMR1* CGG repeats. The normal CGG allele was successfully detected in the PBs (F2-C1-1-PB2, F2-C2-2-PB1, and F2-C2-2-PB2) by GC-rich PCR. Although the mutant allele’s repeat sizes were not detected in the samples (F2-C1-1-PB1 and F2-C2-2-PB1), the results of TP-PCR revealed the expansion statuses (Fig. 3B). It was reported that TP-PCR reliably detected *FMR1* CGG expansion mutations in single cells biopsied from Day 3 embryos [28]. Our study confirmed the efficacy of TP-PCR in PB samples.

The PB-based PGT-M strategy does have some limitations. First, it cannot detect paternal mutations. Second, deducing the genotype of an embryo by detecting PBs assumes that the chromatin containing the disease-causing genes replicates and separates smoothly during meiosis. These chromosomes’ aneuploidy will make detection difficult. Third, when compared to biopsy methods used for cleavage- or blastocyst-stage embryos, the PB-based strategy necessitates more manual interventions and the testing of additional samples. Furthermore, evidence shows that blastocyst-stage embryo transfer results in higher clinical pregnancy and live birth rates than cleavage-stage embryo transfer [29]. Further clinical trials are needed to elucidate whether the PB-based strategy is more beneficial than TE biopsy for women with a small number of oocytes undergoing PGT-M for maternal mutations.

## Conclusions

We conducted PGT-Ms on three couples in which the women carried pathogenic mutations and had reduced oocyte reserves, using a novel PB-based testing approach. Two of these couples successfully gave birth to unaffected offspring. Our findings showed that the PB-based strategy is both feasible and effective for detecting the mutation-carrier statuses of embryos in PGT-M for maternal mutations and alternative to TE biopsy in saving more genetically unaffected embryos. Further clinical trials are needed to determine whether PB biopsy is more beneficial than TE cell biopsy for women with disease-causing mutations and a limited number of oocytes in PGT-M.

## Data Availability

The data of this study are available from the corresponding author on reasonable request. The data are not publicly available due to privacy or ethical restrictions.

## Abbreviations

TE: Trophectoderm
PGT: Preimplantation genetic testing
PGT-M: Preimplantation genetic testing for monogenic disorders
PB: Polar body
PB1: The first polar body
PB2: The second polar body
MDA: Multiple displacement amplification
ICSI: Intracytoplasmic sperm injection
BMI: Body mass index
WES: Whole exome sequencing
TP: Triplet-primed
SNP: Single nucleotide polymorphism
WGA: Whole genome amplification
FET: Frozen-thawed embryo transfer
VWS: Van der Woude Syndrome
FSH: Follicle-stimulating hormone
AMH: Anti-mullerian hormone
HED: Hypohidrotic ectodermal dysplasia
ADO: Allele drop-out
